# Emerging role of LOXL2 in the promotion of pancreas cancer metastasis

**DOI:** 10.18632/oncotarget.9918

**Published:** 2016-06-07

**Authors:** Joon Seong Park, Ji-hae Lee, Yun Sun Lee, Jae Keun Kim, Seung Myung Dong, Dong Sup Yoon

**Affiliations:** ^1^ Pancreatobiliary Cancer Clinic, Department of Surgery, Gangnam Severance Hospital, Yonsei University, Seoul, Korea; ^2^ Research Institute and Hospital, National Cancer Center, Goyang, Gyeonggi, Korea

**Keywords:** pancreas cancer, metastasis, EMT, LOXL2, prognosis

## Abstract

Lysyl oxidase-like 2 (LOXL2) is associated with invasiveness and metastasis in cancer. We analyzed the prognostic impact of LOXL2 in pancreatic cancer patients and investigated the role of LOXL2 in pancreatic cancer cell lines. Immunohistochemical analysis was performed in samples from 80 patients and showed LOXL2 expression in 81.2% of patients with pancreatic cancer. Regarding recurrence patterns, LOXL2-positive tumors showed a significantly higher rate of distant recurrence. The 1-year and 3-year disease-free survival rates were 84.6% and 0.0%, respectively, for LOXL2-negative patients, and 27.8 % and 0.0 %, respectively, for LOXL2-positive patients. On univariate analysis, combined resection of major vessels, depth of invasion, tumor stage, and LOXL2- positive status were significant factors for poor prognosis. After identification of LOXL2 expression in pancreatic cancer cell lines, LOXL2-silenced and LOXL2-overexpressed cell lines were used to perform transwell invasion and transendothelial migration assays.

*In vitro* studies indicated that LOXL2 silencing in MIA PaCa-2 and PANC-1 cells induced a mesenchymal–epithelial transition (MET)-like process associated with decreased invasive and migratory properties. LOXL2 overexpression in AsPC-1 and BxPC-3 cells enhanced the epithelial-mesenchymal transition (EMT)-like process and increased migratory and invasive activity. These clinical and preclinical data confirm that higher LOXL2 expression is associated with the invasiveness of pancreatic cancer cells and the low survival rate of pancreatic cancer patients. Our results suggest the clinical value of LOXL2 as a therapeutic target in pancreatic cancer.

## INTRODUCTION

Pancreatic cancer is one of the most aggressive and lethal malignancies. Although pancreatic cancer is a relatively rare disease, it is the fourth leading cause of cancer-related mortality in Korea. Despite the availability of several treatment modalities, the 5-year survival rate is reported to be lower than 5% [1]. This poor clinical prognosis is mainly associated with early local invasion and a high incidence of recurrence, both of which are characteristic of the disease. To improve the outcomes of pancreatic cancer, the underlying metastatic mechanisms should be better understood.

LOX-like 2 (LOXL2) is a member of the lysyl oxidase (LOX) family, which contains five homologs, LOX and four LOX-like proteins (LOXL1-4), which are secreted, copper-dependent amine oxidases [2–5]. LOXL2 catalyzes the covalent cross-linking of collagen and elastin component side chains through its lysyl oxidase activity, thereby stabilizing the proteins in the extracellular matrix (ECM). Additionally, several studies have shown that LOXL2 down-regulates E-cadherin expression and promotes epithelial-mesenchymal transition (EMT) [6–8]. Due to these mechanisms, LOXL2 is associated with aggressive cancer characteristics and poor patient prognosis. Several studies have reported that LOXL2 is associated with poor prognosis in cancer patients [9–13], and that overexpression of LOXL2 promotes invasion activity among cancer cells [10–12, 14, 15]. LOXL2 is up-regulated in human pancreatic cancer, and might be correlated with the regulation of different transcription factors associated with invasion and metastasis [16]. The activity of lysyl oxidase (e.g., LOX and LOXL2) also, correlates with oncogenic stress response and tumorigenesis in pancreatic ductal adenocarcinoma [17]. However, there is a paucity of clinical evidence regarding the role of LOXL2 and its functions in pancreatic cancer.

In this study, we aimed to confirm the role of LOXL2 in the increased invasiveness of pancreatic cancer cells and to evaluate the clinical impact of LOXL2 in patients with pancreatic cancer. To accomplish this, we examined pancreatic cancer tissue for LOXL2 expression via immunohistochemical (IHC) staining and evaluated the prognostic significance of LOXL2 for pancreatic cancer patients. Moreover, we performed *in vitro* studies that showed an association between an aggressive cancer prognosis and LOXL2 expression.

## RESULTS

### Clinicopathologic characteristics

The study involved patients of a mean age of 63.3 (± 9.7) years including 39 men and 41 women. Of the 80 patients, eight underwent pancreaticoduodenectomy, 41 underwent pylorus-preserving pancreaticoduodenectomy, 26 underwent distal pancreatectomy with splenectomy and five underwent total pancreatectomy. According to the 7^th^ AJCC classification, one patient was stage IA, four were stage IB, 22 were stage IIA, 50 were stage IIB, and three were stage III. All patients received adjuvant chemotherapy with six cycles of gemcitabine every 4 weeks. Each chemotherapy cycle consisted of three weekly infusions of gemcitabine 1000mg/m^2^ administered via intravenous infusion over a 30-min period, followed by a 1-week pause.

### Analysis of clinicopathologic features and LOXL2 status

Among the 80 patients, 65 (81.2%) were positive for LOXL2. The clinicopathologic features and LOXL2 status of patients in the study group are summarized in Table 1. There was no statistical difference in clinicopathologic characteristics according to LOXL2 status.

**Table 1:** Patients characteristics based on LOXL2 status

	LOXL2 negative (N=15)	LOXL2 positive (N=65)	*p-value*
Age (mean ± SD), yr	63.1 ± 10.9	63.3 ± 9.4	*0.961*
Sex			*0.858*
Male	7	32	
Female	8	33	
Operative Procedure			*0.186*
PD/PPPD	7	42	
Distal pancreatectomy	8	18	
Total pancreatectomy	0	5	
Combine resection			*0.551*
No	13	52	
Yes	2	13	
Tumor size (mean ± SD), cm	3.5 ± 1.6	3.4 ± 1.8	*0.782*
Histologic grade			*0.138*
Well differentiation	2	12	
Moderate differentiation	8	45	
Poor differentiation	5	8	
Depth of invasion			*0.713*
T1	0	1	
T2	2	5	
T3	13	56	
T4	0	3	
Lymph node metastasis			*0.881*
Negative	5	23	
Positive	10	42	
Perineural Invasion			*0.671*
No	4	21	
Yes	11	44	
Lymphovascular invasion			*0.775*
No	7	33	
Yes	8	32	
Tumor Stage			*0.461*
IA	0	1	
IB	1	3	
IIA	4	18	
IIB	10	40	
III	0	3	

### Patterns of recurrence according to LOXL2 status

Among the 80 patients, 67 (83.8%) had tumor recurrence confirmed via pathologic or radiologic examination. Regarding recurrence patterns, LOXL2-positive tumors showed a significantly higher rate of distant recurrence (Table 2).

**Table 2:** Patterns of recurrence according to LOXL2 status

	LOXL2 negative	LOXL2 positive	*P-value*
Recurrence patterns			*0.044*
Local recurrence	8	17	
Distant recurrence	5	37	

### Prognostic impact of LOXL2 status

**Figure 1:**
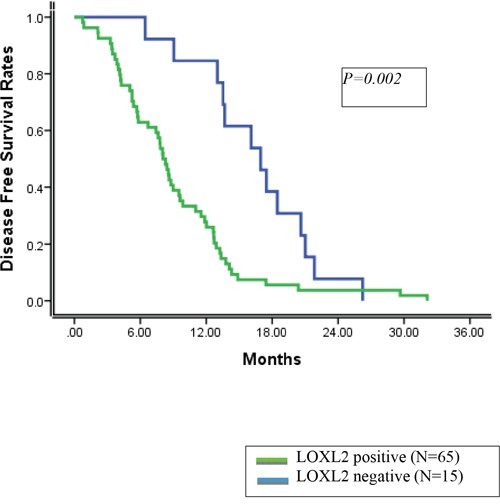
Disease free survival rates according to LOXL2 status

In these 80 patients, the 1- and 3- year disease-free survival (DFS) rates were 38.8 % and 0.0%, respectively. On univariate and multivariate analyses, tumor stage and LOXL2-positive status were identified as independent prognostic factors for DFS (Table 3, 4). The 1 and 3-year DFS rates were 84.6% and 0.0%, respectively, for patients with LOXL2-negative tumors and 27.8% and 0.0%, respectively, for patients with LOXL2-positive tumors (Figure 1).

**Table 3:** Univariate prognostic factors of disease free survival rate in pancreatic cancer

	1-yr DFS	3-yr DFS	*p-value*
Age (yr)			*0.956*
≥ 60	40.4	0.0	
< 60	35.0	0.0	
Sex			*0.152*
Male	51.5	0.0	
Female	26.5	0.0	
Operative Procedure			*0.190*
PD/PPPD	26.2	0.0	
Distal pancreatectomy	60.0	0.0	
Total pancreatectomy	60.0	0.0	
Combine resection			*0.876*
No	37.5	0.0	
Yes	45.5	0.0	
Tumor size (cm)			*0.892*
≥ 2cm	39.3	0.0	
< 2cm	40.0	0.0	
Histologic grade			*0.373*
Well differentiation	54.5	0.0	
mod differentiation	31.8	0.0	
poor differentiation	50.0	0.0	
Depth of invasion			*0.717*
T1/T2	40.0	0.0	
T3/T4	38.7	0.0	
Lymph node metastasis			*0.240*
Negative	52.2	0.0	
Positive	31.8	0.0	
Perineural Invasion			*0.318*
No	34.8	0.0	
Yes	40.9	0.0	
Lymphovascular invasion			*0.4111*
No	33.3	0.0	
Yes	44.1	0.0	
Tumor Stage			*0.033*
IA/IB	33.3	0.0	
IIA	61.1	0.0	
IIB	32.6	0.0	
III	0.0	0.0	
LOXL2 status			*0.002*
Negative	84.6	0.0	
Positive	27.8	0.0	

**Table 4:** Univariate analysis of disease free survival rate in pancreatic cancer

Variables	P-values	Odds ratio	Confidence interval (95%)	
			Lower	Upper
LOXL2 positive	0.002	2.810	1.474	5.357
Tumor stage	0.047	1.623	1.007	2.618

The overall 3-and 5-year survival rates were 20.0% and 6.1%, respectively. On univariate analysis, combined resection of major vessels, depth of invasion, tumor stage and LOXL2-positive status were significant factors for poor prognosis. In the multivariate analysis, combined resection of major vessels was identified as an independent prognostic factor for overall survival.

### LOXL2 expression and invasiveness in pancreatic cancer cell lines

**Figure 2:**
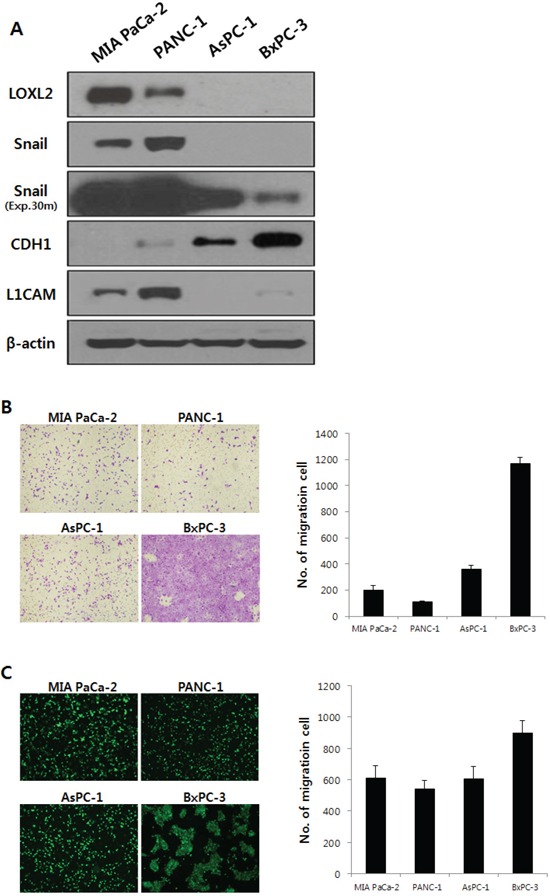
LOXL2 expression and properties of migration and invasiveness were analyzed in pancreatic cancer cell lines **A.** on Western blot analysis, LOXL2 was only detected in MIA PaCa-2 and PANC-1. Exp.30m, Snail was analyzed after 30 minutes of exposure. Each cell showed independent properties of migration and invasiveness (**B.** transwell invasion assay; **C.** transendothelial migration assay).

Expression of LOXL2 was analyzed in a series of pancreatic cancer cell lines: MIA PaCa-2, PANC-1, AsPC-1, and BxPC-3. LOXL2 was detected in MIA PaCa-2 and PANC-1 yet, not in AsPC-1, and BxPC-3 (Figure 2A). LOXL2 expression correlated with the expression of Snail and L1CAM and was inversely proportional to the expression of epithelial cell markers such as CDH1 (Figure 2A). An analysis of transwell invasion (Figure 2B) and transendothelial migration (Figure 2C) indicated that expressions of these genes were not correlated with the properties of migration and invasiveness of each pancreatic cancer cell, suggesting that each pancreatic cancer cell has LOXL2- independent mechanisms to control the expression of LOXL2, Snail, CDH1, and L1CAM, which is related to EMT and invasiveness.

### The effects of LOXL2 expression on invasiveness

**Figure 3:**
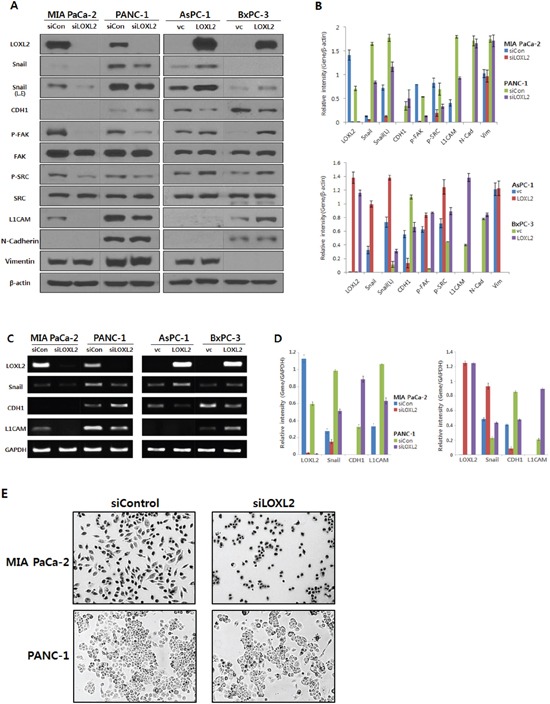
Analysis of LOXL2 expression in pancreatic cancer cell lines with LOXL2-silencing and LOXL2-overexpression **A.** Western analysis and **B.** densitometric analysis using NIH image; LOXL2 silencing (siLOXL2) reduced LOXL2, Snail, p-FAK, p-SRC and L1CAM, and increased CDH1 in MIA PaCa-2 and PANC-1. LOXL2 overexpression (LOXL2) showed increased LOXL2, Snail, p-FAK, p-SRC and L1CAM, and reduced CDH1 in AsPC-1 and BxPC-3. **C.** RT-PCR analysis and **D.** quantitation of RT PCR; LOXL2 silencing has an effect on the reduction of LOXL2, Snail, and L1CAM, and on the increase of CDH1. LOXL2 overexpression has an effect on the increase of LOXL2, Snail, and L1CAM, and on the reduction of CDH1. **E.** MIA-PaCa-2-siLOXL2 cells exhibited a more epithelial phenotype than si-control. siCon, siRNA control using siRNA scramble; siLOXL2, siRNA for LOXL2; vc, vector(pc3.1) control; LOXL2, pc3.1-LOXL2; L.E, Snail was exposed for more 3 min more than other proteins.

**Figure 4:**
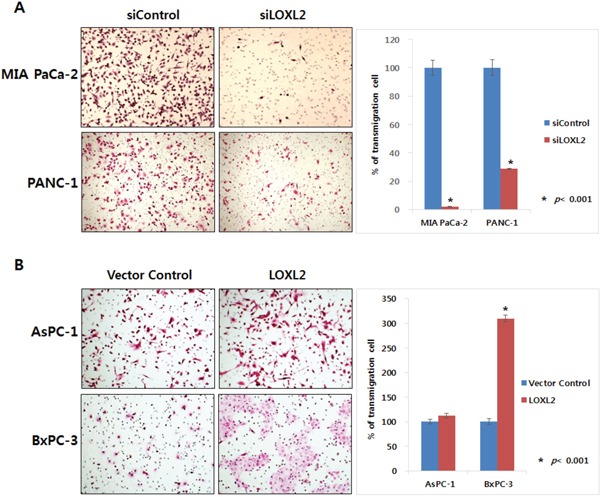
Effect of LOXL2 on pancreatic cancer cell invasion based on transwell invasion assay LOXL2-silencing **A.** and LOXL2-overexpression **B.** have an effect on the capacity of motility and invasiveness of pancreatic cancer cell lines.

**Figure 5:**
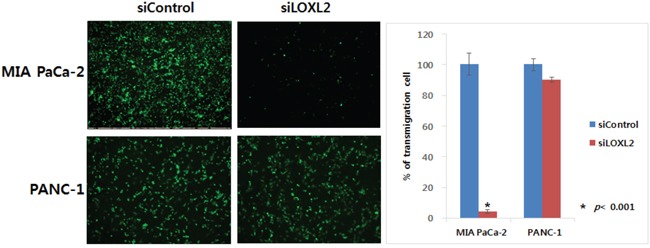
Effect of LOXL2 on pancreatic cancer cell invasion based on transendothelial migration assay LOXL2-silencing has an effect on the capacity of motility and invasiveness of MIA PaCa-2 and PANC-1.

We generated stable pancreatic carcinoma cells in which LOXL2 was silenced by siLOXL2, (MIA PaCa-2 and PANC-1) or ectopically expressed by pc3.1- LOXL2 (AsPC-1 and BxPC-3). Selected clones from MIA-PaCa-siLOXL2 and PANC1-siLOXL2 showed significantly reduced LOXL2 protein (Figure 3A and 3B) and transcript levels (Figure 3C and 3D). These cells exhibited significant changes in the expression of molecules related to invasiveness and EMT. LOXL2 silencing induced significant correlation between reduced Snail expression and increased CDH1 expression in PANC-1-siLOXL2 cells, though, not in MIA PaCa-2 cells. Considering that CDH1 protein was not detected in MIA PaCa-2 due to its transcriptional silencing (Figure 3A and 3B) and the low levels of CDH1 expression (MIA PaCa-2) and downregulation (MIA PaCa-2-siLOXL2), LOXL2 silencing and reduced Snail might not affect the re-expression of CDH1. The expressions of vimentin and N-cadherin, which are related to EMT, were not affected significantly by LOXL2, as demonstrated by slight up-regulation of these proteins on densitometric analysis of NIH images (Figure 3B). Downregulation of phospho-SRC and phospho-FAK was detected on Western blot analyses and reduced expressions of Snail and L1CAM were detected at both mRNA and protein levels (Figure 3A-3D). Contrary to a previous report by Rückert et al. [16], our experimental set for LOXL2 knockdown by siLOXL2 influenced the cell morphology, resulting in cells that were less spindle-shaped, less atypical, and smaller, though only in MIA-PaCa-2-siLOXL2; however, silencing or ectopic overexpression of LOXL2 did not affect the morphology of the other cell lines (Figure 3E). Importantly, silencing of LOXL2 resulted in a marked decrease in motility and the invasiveness capacity of MIA PaCa-2 (p < 0.001) and PANC-1 cells, as determined from transwell invasion and transendothelial migration assays, respectively (Figure 4A and 5).

We also observed a significant influence of ectopic overexpression of LOXL2 in AsPC-1-LOXL2 and BxPC-3-LOXL2 cells. Decreased expression of CDH1 and increasing phospho-FAK / phospho-SRC were observed in AsPC-1-LOXL2 and BxPC-3-LOXL2 cells (Figure 3A-3D). Upregulation of L1CAM was detected only in BxPC-3-LOXL2. Upreulation of Snail expression was also detected in both AsPC1-LOXL2 and BxPC3-LOXL2. (Figure 3A-3D). Conversely, LOXL2 overe-xpression enhanced the migration potential of BxPC-3-LOXL2 (p < 0.001), but not AsPC-1-LOXL2. (Figure 4B).

## DISCUSSION

LOXL2, a member of the LOX family, is responsible for the stabilization of collagen and elastin fibers in ECM through covalent oxidative deamination. Tissue fibrosis is associated with cancer progression through direct promotion of cellular transformation and metastasis [18]. Lu et al. reported that the increased collagen cross-linking in mouse mammary stroma induced by LOXL2 activity is associated with ECM stiffness and tumor invasion and progression [14]. These studies are in agreement with the notion that tumor fibrosis increases the invasive behavior of tumors by activating LOXL2 signals and suggest that LOXL2 is involved in cell adhesion, cell migration and invasion, and EMT transformation [6, 9].

Although many studies have shown that increased LOXL2 expression is associated with lymph node metastasis, lymphatic invasion, and advanced tumor stage [10, 11], IHC staining of LOXL2 was not associated with clinicopathologic variables in this study. This disparity might be explained by the unusual characteristics of pancreatic cancer, which exhibits a high expression of EMT properties even in its early stage. In fact, the ratio of LOXL2 positivity in early-stage pancreatic cancer was higher than that in breast cancer, renal cell carcinoma, and stomach cancer [10, 11, 19]. This disparity necessitates further investigation of LOXL2 in pancreatic cancer. Moreover, investigation of recurrence patterns showed that LOXL2 expression was significantly associated with distant metastasis, including the liver and lung, as described in a previous report [10]. This result suggests that LOXL2 expression might be associated with the EMT activity of pancreatic cancer. On univariate and multivariate analyses of DFS, the prognosis of LOXL2-positive patients was significantly worse than that of LOXL2-negative patients. These findings suggest that LOXL2 over-expression might be a poor prognostic factor in pancreatic cancer, based on evidence that LOXL2 expression in pancreatic cancer cells might contribute to metastasis in a clinical setting, and further suggest that inhibition of LOXL2 might provide a survival benefit to patients with pancreatic cancer.

To identify whether the clinical significance of LOXL2 expression in pancreas cancer patients is associated with metastasis and EMT properties, we conducted an *in vitro* study of LOXL2 in pancreatic cancer cells.

In previous studies, intracellular functions of LOXL2 have been postulated relating to the promotion of EMT and the invasiveness of cancer cells. LOXL2 induces EMT by stabilizing Snail, a suppressor of CDH1 [8, 20, 21] in carcinoma progression [6]. LOXL2 even controls CDH1 by regulating H3K4me3 deamination [22], and LOXL2-E47 EMT factor plays a role in the repression of CDH1 in early metastasis colonization of breast cancer cells [23]. Additionally, positive modulation of the FAK and SRC pathways by LOXL2 has been reported, which can contribute to cell migratory behavior [8, 11, 20, 24, 25]. More recently, Holly E. Baker et al. showed that LOXL2 activated fibroblasts via integrin-mediated FAK activation in tumor cell invasion and metastasis [26]. The expression of L1CAM, an adhesion molecule, is associated with poor prognosis and spontaneous metastasis of cancer cells to the lung [27–29]. Additionally, EMT and NF-κB activation is associated with the up-regulation of L1CAM, which enhances cell invasion and motility and tumor metastasis formation [30]. Gregg et al. suggested that hypoxia inducible factor dependent on expression of L1CAM and LOX family members (LOX, LOXL2, and LOXL4), which promotes cancer cell extravasation and metastatic niche formation in breast cancer cells [31].

We found that LOXL2 plays an integral role in the promotion of EMT and the invasiveness of pancreatic cancer cells. Our *in vitro* study indicated that LOXL2 activates EMT-like processes in pancreatic cell lines that are associated with invasive and migratory properties. Moreover, LOXL2 contributes positively to the activation of FAK/SRC and influences the expressions of CDH1, Snail and L1CAM, which are all related to EMT and the invasiveness of pancreatic tumor cells [12, 13, 15, 29, 32–35]. The present results support the notion that LOXL2 is involved in the maintenance of the mesenchymal phenotype in pancreatic cancer cells and are consistent with previous results in other studies [25, 36–39].

It has been reported that LOXL2 is one of the most highly and specifically upregulated genes in pancreatic cancer, compared to normal pancreatic tissues [40]. Proteomic profiling detected the gene product of LOXL2 in pancreatic cancer cells as well as in the secretions of pancreatic cancers [41, 42]. In addition, LOXL2 has been associated with pancreatic cancer pathology and tumorigenicity. For instance, inhibition of LOXL2 has been shown to result in the reduced viability of pancreatic cancer cells and their increased sensitivity to chemotherapy [16]. Moreover, in light of the promising role of LOXL2 in promoting the activation of EMT-related molecules that increase the aggressiveness of pancreatic cancer, it is noteworthy that LOXL2 was prominently expressed in the majority of pancreatic cancer tissues in our study.

Our finding has clinical importance in that provides a novel potential therapeutic target for pancreatic cancer. Based on experimental evidence, LOXL2 has previously been proposed as a therapeutic target in the treatment of pancreatic cancer [23, 43, 44]. The current targeting strategy for LOXL2 focuses on inhibiting its enzymatic activity. LOXL2 seems to be effectively targeted at the protein level either through the use of small-molecule inhibitors that may act both intracellularly and extracellularly, or through the use of antibodies [44]. Therefore, our data also support a therapeutic approach in which intracellular LOXL2 expression could be an effective target molecule for the improved survival of pancreatic cancer patients.

Although there were limitations to our study, including the retrospective study design and small sample size, our findings provide preclinical and clinical evidence that the contribution of LOXL2 enzymatic activity to metastasis in an experimental setting can be translated into poor survival outcomes in patients with pancreatic cancer.

In conclusion, results from an IHC analysis of tumors demonstrated that LOXL2 is an independent marker for metastatic disease and death in patients with pancreatic cancer. Additionally, our *in vitro* study demonstrated that LOXL2 expression promotes EMT and the invasiveness of pancreatic cancer cell lines, a finding compatible with the results of previous *in vitro* studies. These findings suggest that LOXL2 could potentially be a valuable target for the improvement of survival rates in patients with pancreatic cancer.

## MATERIALS AND METHODS

### Patients

Between June 2002 and December 2012, 84 patients underwent radical curative resection for pancreatic cancer at Gangnam Severance Hospital, Yonsei University College of Medicine, Seoul, Korea. Among these patients, four were excluded due to poorly preserved tissue samples, incomplete clinicopathologic data, or loss to follow-up. As a result 80 patients who had undergone curative resection were retrospectively reviewed. All patients were followed up for more than 6 months and the mean duration of follow-up was 33.2 ± 9.7 months. Patients were followed every 3 months during the first 12 months, and then every 6 months beyond the first year. This study was approved by the Institutional Review Board of Gangnam Severance Hospital, Yonsei University, Seoul, Korea (3-2014-0153).

### Immunohistochemical (IHC) Staining

Serial sections (5 μm) of each block were adhered to poly-L-lysine covered slides and incubated at 62°C for 60 minutes. After the elimination of paraffin with xylene and staged ethyl alcohol dehydration, the sections were heated in a microwave containing a 10-mM citrate buffer (pH 6.0) solution for 15 minutes. The primary antibody against LOXL2 (Abcam, Cambridge, UK) was used at a 1:1,000 dilution according to the manufacturer's instructions. The procedure has been described in detail elsewhere [21]. Normal pancreas tissue present within the block and appropriate control tissues were used as positive controls.

**Figure 6:**
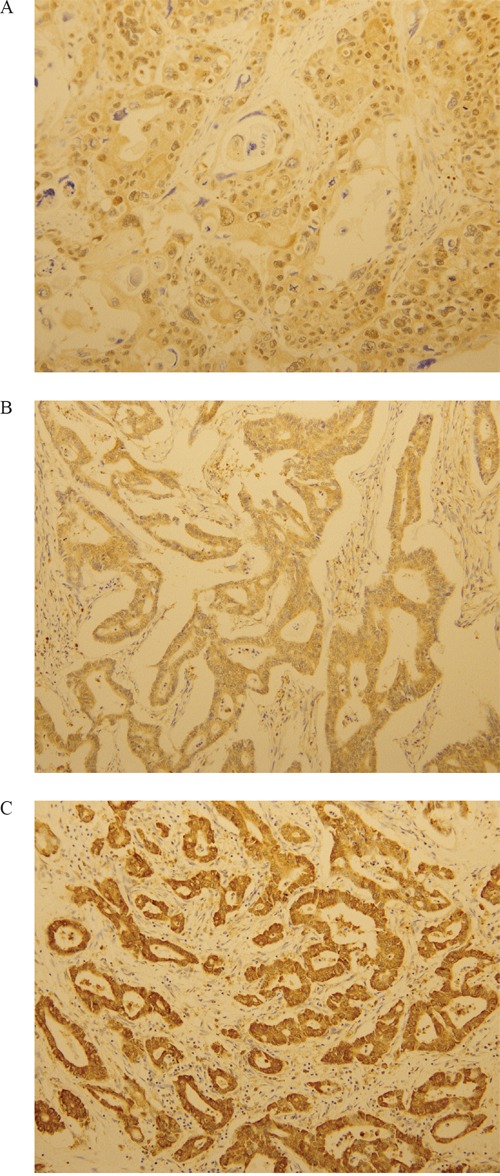
Immunohistochemical analysis of LOXL2. LOXL2 expression was evaluated at a high-power field (x200 magnification) **A.** One positive for LOXL2. **B.** Two positives for LOXL2. **C.** Three positives for LOXL2.

IHC staining for LOXL2 was categorized as negative, “1+”,“2+”, or “3+” in high-power fields (200x magnification) according to the intensity of cytoplasmic staining (Figure 6). LOXL2-positive status was assigned for scores “2+”and “3+”. The interpretation of IHC was evaluated by two pathologists who had no information regarding the clinical outcomes.

### Cell culture

Pancreatic cancer cell lines MIA PaCa-2, PANC-1, AsPC-1, and BxPC-3 were purchased from the American Type Culture Collection (ATCC; Manassas, VA, USA) and grown in accordance with ATCC recommendations. MIA PaCa-2, PANC-1 and AsPC-1 were poor differentiation cell type, and BxPC3 is moderate to poor differentiation cell type [45]. MIA PaCa-2 and PANC-1 were characterized as LOXL2-positive whereas AsPC-1 and BxPC3 were characterized as LOXL2-negative pancreatic cancer cells.

### Construction of LOXL2-siRNA and LOXL2 expression vector

For knockdown of LOXL2 mRNA, we used the following sequences: siLOXL2, 5′-CAGUCUAUUAUAGUCACAU-3′. As a negative control, we used siRNA targeting green fluorescence protein: 5′-GGUGUGCUGUUUGGAGGUCTT-3′. The human LOXL2 cDNA clone was provided from the Korea Human Gene Bank, Medical Genomics Research Center, KRIBB, Korea. The LOXL2 clone was inserted into the mammalian expression vector pcDNA3.1(+) (Invitrogen, San Diego, CA) by PCR using the following primers: (F: 5′- CTA GCT AGC ATG GAG AGG CCT CTG-3′ and R: 5′- CGC GGA TCC TTA CTG CGG GGA CAG -3′). The construct was verified by sequencing. Cells were transfected with LOXL2-siRNA or pc3.1-LOXL2 at 50 % confluence using the transfection reagent Oligofectamine (Invitrogen, Carlsbad, CA, USA), according to the manufacturer's instructions.

### Reverse transcription polymerase chain reaction (RT-PCR) and quantitative RT-PCR

RNA was extracted using the Trizol reagent (Invitrogen, Carlsbad, CA), and cDNA was synthesized using the MMLV enzyme (Invitrogen). PCR was performed at 95^°^C for 10 min and in 25 cycles at 95^°^C for 15 s, 62^°^C for 30 s, and 72^°^C for 30 s on a GeneAmp PCR System 9700 (Applied Biosystems, Foster City, CA, USA). Each PCR product was analyzed in 20% agarose gel. Quantitative RT-PCR was performed on LightCycler^®^480 Real-Time PCR System (Roche Diagnostics, Mannheim, Germany) using SYBR Green I Matermix (Roche Diagnostics, Mannheim, Germany). Primers used for RT-PCR were LOXL2 (F: 5′-AACGAGGCGACCCTTGCAGC-3′ and R: 5′- GGGTGCGCTTGCGGTAGGTT-3′); Snail, (F: 5′-AATCGGAAGCCTAACTACAGCGAG-3′ and R: 5′-CTTTCCCACTGTCCTCATCTGACA-3′); CDH1, (F: 5′-GACGCGGACGATGATGTGAAC-3′and R: 5′-TGTACGTGGTGGGATTGAAGA-3′); L1CAM, (F: 5′-GCCACCTGTCATCACGGAAC-3′ and R: 5′-GTCCAGCGGAACTGCACTTC-3′) and GAPDH, (F: 5′-CGGGAAGCTTGTGATCAATGG-3′ and R: 5′-GGCAGTGATGGCATGGACTG-3′). The experiments were performed in triplicate and normalized to GAPDH.

### Western blot analysis

Cell lysates were prepared in RIPA buffer (Sigma, St Louis, MO, USA) supplemented with protease inhibitors. Protein samples were separated by 10% sodium dodecyl sulfate–polyacrylamide gel electrophoresis and transferred onto nitrocellulose membranes. The membranes were blocked and probed with primary antibody against LOXL2 (Origene; Rockville, MD, USA), Snail (Cell Signaling; Danvers, MA, USA), pFAK and FAK (Santa Cruz, CA, USA), pSRC (Millipore, Billerica, MA, USA), SRC (Santa Cruz, CA, USA), CDH1 (BD Biosciences; Sparks, MD), L1CAM (Novus Biologicals, Littleton, CO, USA), N-cadherin (Invitrogen, CA, USA), Vimentin (Dako, Glostrup, Denmark) and β-actin (Sigma, St Louis, MO, USA). The intensity of the western blotss was quantified by densitometric scanning with ImageJ (NIH) and normalized to β-actin.

### Tranwell invasion assay

The invasive potential of pancreatic cancer cells was assessed *in vitro* in matrigel-coated invasion chambers (Corning; NY, USA) in accordance with the manufacturer's instructions. Briefly, cells in the log growth phase were serum starved for 24 h prior to seeding, detached by brief trypsinization and resuspended in medium containing the appropriate treatment. The matrigel invasion inserts were rehydrated and prepared as described in the manufacturer's instructions. Cells in serum-free medium at a density of 1 × 10^5^ cells/ml/well were placed on the top of matrigel-coated polycarbonate filters (8 μm pore size) suspended in a membrane invasion culture system chamber; the chamber underneath the membrane contained complete medium. The cells were incubated in a CO2 incubator at 37^°^C for 5 h, after which the non-invasive cells were removed from the upper surface of the membrane and the invasive cells on the undersurface of the membrane were fixed and stained with hematoxylin–eosin (H&E; Sigma-Aldrich). These experiments were performed in triplicate at least three times. The invading cells were counted under a fluorescence microscope in five random high-power fields.

### Transendothelial migration assay

HUVEs and EGM^TM^-2 media (Lonza, Walkersville, MD, USA) was purchased. HUVECs (3×10^4^ in 200 μl of EGM-2 medium) were seeded onto 0.1% gelatin-coated inserts in the transwell chamber (8-μm pore size and 6.5-mm diameter) and allowed to form a monolayer for 48 h. The endothelial culture medium was removed from each insert and CFSE-labeled cancer cells (1×10^5^ cells in 200μl of serum-free medium) were added on top of the HUVEC monolayer. Cancer cell culture medium containing 10% FBS was added to the lower chamber. After incubation for 48 or 72 h, non-migratory cells were wiped off with cotton swabs and the membrane was washed with PBS. Finally, the membranes were removed from the transwell insert, mounted onto slides and observed under a fluorescence microscope at ×10 magnification.

### Statistical analysis

All clinicopathologic variables except age and tumor size were used as categorical variables. Differences in continuous variables between the two groups were evaluated using Student's t-test, and differences in categorical variables were evaluated by the chi-square test. The Kaplan-Meier method was used to calculate and display survival curves, and the log-rank test was performed to determine differences among all groups. Overall survival was defined as the time interval between the date of surgery and the date of death from cancer or the last follow-up. Recurrence-free survival was defined as the time interval between the date of surgery and the date of recurrence or last follow-up. The Cox proportional hazards regression method was used to determine independent prognostic factors. A P-value of less than 0.05 was considered to be statistically significant.
